# Hospital and Institutional News

**Published:** 1913-03-08

**Authors:** 


### HOSPITAL AND INSTITUTIONAL NEWS.

### A HOSPITAL DISPUTE IN IRELAND.

As we go to press it is announced by a circular letter dated March 1, and published by the consulting staff of the Royal National Hospital for Consumption for Ireland, at Newcastle, County Wicklow, that the whole honorary medical staff, including the consulting surgeon, have resigned. It appears that a disagreement took place last December between the board of management and the consulting staff regarding the board's proposal to appoint an outside medical man to try his special treatment of tuberculosis at the hospital, where two wards were to be set apart for this purpose. It was announced eventually that this medical man had been appointed as an additional physician. Conferences between the board and the honorary medical staff followed, and the latter now announce their resignation as the meetings failed to result in an agreement. No hasty judgment must be formed, for such disputes are far too easily labelled as an "example of medical jealousy," or "an instance of undue influence in staff appointments." Both parties to the dispute, if they are wise and have the best interests of the hospital at heart, will publish their case. For this is the only way by which hasty judgment and consequent bitterness can be avoided.

### SIR ARTHUR DOWNES ON ADMINISTRATION.

The whole Poor-Law infirmary world is indebted to Sir Arthur Downes for his very remarkable speech at the opening of the new nurses' home at the Southwark Infirmary. From the Senior Medical Officer for the Poor-Law Department of the Local Government Board one may hope for a thoroughly well-informed speech, but Sir Arthur's differed from most official speeches in that he did not labour to conceal his knowledge, which it is so often the temptation of official heads to do. After reminding his hearers that "at the present day there are more beds in the Poor-Law infirmaries in London alone than in all the general hospitals in England and Wales," he uttered some pregnant remarks on the secret of successful administration. We give the secret in his own words: "I am very glad to hear of the good relations which exist between the Guardians and the medical and nursing staff. The first principle of administration in an institution like yours is to get good officers, and, having proved them to be such, to trust them." The Hospital has often pointed out, for instance, in connection with the Draft Poor-Law Order, that the difficulties and bad blood which undoubtedly still exist in the smaller unseparated infirmaries are due, first, to parsimony of a system which will not try to make the lower posts of the institutional Poor-Law medical service attractive to the rest of the junior men; and, secondly, to the fact that the men who are content—however unwillingly—to apply for the junior posts, *ex hypothesi* are not of such energy as to win independence for their administration, the conditions being such as to damp—and, indeed, often to destroy—all but the ardour of genius. The Draft Poor-Law Order was an attempt to ameliorate the evils necessitated by a bad system. The secret of a good one is made evident by Sir Arthur Downes' speech. We wish the above passage could be printed in six-inch letters and hung, by law, in the board room of every Board of Guardians in the country.

### AN EX-M.P. AND INSURANCE CONTRIBUTIONS.

Captain Bryan Cooper, ex-Member of Parliament for South County Dublin, has been fined for having failed to pay Insurance contributions in respect of thirteen indoor servants. The Captain's butler pointed out that his employer paid a doctor to attend his servants, and that their wages were always paid during illness. He added the insignificant fact that the servants of their own accord refused to present their cards for stamping, but that Captain Cooper's instructions were to stamp any cards which might be presented for the purpose. Captain Cooper remarked that he gave his servants benefits equal, if not superior, to those of the Insurance Act, and had done so long before the Act was passed. In addition to the benefits to which his butler drew attention, he had paid half the cost of a shelter in Sligo, built by the Women's National Health Association. He told his servants that they could decide as they pleased, and he was quite willing to pay his share under the Act. His attitude is defined quite clearly by his further statement that he hoped such cases as his would result in an amending Act. While imposing a small fine the Bench regarded him as a model employer, and refused an application for professional costs. This is the clearest instance which we have yet come across of the true cause of the trouble. We have official testimony to the character of the employer, to the free choice allowed by him to the employed, and to the actual and ungrudging benefits the employed in this occupation have actually enjoyed for years past without the aid of Parliament. No one reading the facts can doubt also the impartiality and justice in law and in ethics of the Bench in the present case. The public spirit of all parties is unexceptional. It is the hated Act and that alone which has produced the ill-feeling, and for its amendment we will ever work and pray.

### NEW SUPERINTENDENT OF ST. ANDREW'S MENTAL HOSPITAL.

More than ordinary interest attaches to the fact that the governors of St. Andrew's Hospital for Mental Diseases, Northampton, have appointed Dr. Frederick Rambaut as medical superintendent in succession to the late Dr. Bayley. Dr. Rambaut is now medical superintendent of Shropshire County Mental Hospital, Shrewsbury. He was educated at Dublin University. At the time when Dr. Bayley's death was announced we drew attention to the long connection of the late superintendent and his son with St. Andrew's Hospital, Dr. Bayley, jun., having been his father's chief assistant for many years. The governors' decision to introduce an officer who has had experience elsewhere is a fact to be noted.

### SANATORIA BUILDINGS AT £50 PER BED.

A very interesting Memorandum, prepared by the Architect and the Medical Officer to the Local Government Board, has been issued on the construction and general possibilities of inexpensive buildings for the treatment of tuberculosis. The general trend of the Memorandum's advice appears to be "for additional accommodation to be provided with expedition, seek an institution where administrative accommodation already exists; for complete new institutions the cheap forms of construction may be recommended." It is also suggested that a sanatorium for the treatment of incipient cases should, if possible, from motives alike of economy and efficiency, contain not less than one hundred beds. Where the population of an area would not justify so large a number, a combination of areas is suggested. It is also laid down that, where a dispensary system is in operation, the selection of suitable cases for institutional treatment can be best carried out. The dispensary thus receives its due. The proposals in the Memorandum, which every institutional worker in the sanatorium or chest-hospital sphere should most certainly study, are illustrated and enforced by an accompanying plan of a pavilion for fifty beds, and of another for twenty beds. These are given to exemplify the two types of construction and buildings which the authors of the memorandum consider generally suitable. Details are added to show that under ordinary conditions the cost of the patients' accommodation, constructed on the timber-framing principle, should not exceed fifty pounds per bed exclusive of the administration expenses. Once again the Local Government Board comes forward as the most practical of Government Departments, the one which is most prepared to face facts, and least afraid of the trouble involved by giving thought, care, and aid in their most practical elucidation.

### TWO NEW DEPARTMENTS AT MIDDLESEX HOSPITAL.

We are glad to note that the Middlesex Hospital continues in the way of luck, and that the publicity-giving schemes, which of late years have made it one of the best advertised of London institutions, have set a fashion of benevolence in its favour. For benevolence has its fashions like everything, and wise is the institution which diverts the tide its own way. The well-known "anonymous donor," who has become a classic figure in contemporary life, has announced his intention to pay the whole cost of a new complete pathological block and institute of hygiene, which will involve drastic alterations to the premises of the school. Of course, the scheme existed before the means to carry it out, and the £10,000 which it is expected to cost will start with every chance of proving a good investment. For the most difficult of all schemes to carry through are those which have, as is not uncommon, to be invented in order that a charitable gift may somehow or other be disposed of. It will be noted, however, that the scheme is twofold, and that part which concerns the proposed institute of hygiene is the more immediately interesting, since the scope and equipment of such a department are connected in the public mind rather with the commercial exploitation by unregistered "practioners" of certain elementary truths such as the value of exercise, than with official hospital treatment.

### RESPONSIBLE POSITIONS AT EARLY AGES.

Medical men seldom, in the nature of things, reach responsible positions in the institutional world at early ages, and hence records of service of half a century or more are extremely rare. At Nottingham, however, the Coppice Hospital for Mental Diseases has for medical superintendent Dr. W. B. Tate, and he has held that position, according to the *Nottingham Guardian*, for fifty-four years. At the recent annual meeting of the governors Dr. Tate was unable to be present, owing to serious illness, and this was the first annual meeting he had missed since his appointment. The main feature of the report was the result of the two inspections by the Lunacy Commissioners in May and December of last year. Both reports were highly satisfactory, and it is especially to be noted that in December not a single patient had any complaint to make. Those who know what curious and ingenious complaints mental patients manufacture for the inquisition of the Commissioners will realise that the Coppice inmates must be a very unusually contented set. The weekly average cost per head is forty-five shillings, towards which all the patients contribute more or less, according to their means. Dr. Tate's first qualification, we may add, dates back to 1849; so that he had been already ten years qualified when he became superintendent at the Coppice; and he can, therefore, hardly be much less than eighty-five years of age. We trust that he will soon be restored to health and vigour.

### RADIO-THERAPY AND PUBLIC PANIC.

It is good to have personal as well as official testimony to the value of the work that is being done at the new Radio-Therapeutic Department of the Cancer Hospital in Fulham Road. At last week's annual meeting the medical committee definitely reported that diagnosis and treatment had been greatly facilitated and that the new department had "enormously increased" the usefulness of the hospital. The director, Dr. Knox, added a useful particularisation to the above by reporting that he was working out with Dr. Salmond's help a complete system of $x$-ray dosage. When the system is completed, or indeed parallel with its completion, he hopes to elaborate a technique. Another point which is engaging the attention of the staff of the new department is the all-important question why some patients respond and other patients fail to respond to treatment. At least it is hoped that light may be thrown upon this question by the investigation of blood-changes occurring in patients undergoing treatment which is being carried on. In addition, of course, the research department, of which Dr. Paine is the head, is also tackling problems which are (unfortunately!) too interesting to be summarised in a Note. Perhaps Dr. Paine will appreciate our reticence by allowing himself a freedom of description in another issue which we have denied to ourselves in this. We are very glad, indeed, to see the annual meeting thus taken advantage of to advertise the living work of the institution at the moment in this way. That indeed is the chief, if not, in a high sense, the only, justification for the annual meeting at all. Mr. Ryall, chairman of the medical committee, evidently understood quite clearly that his true audience was outside the room rather than in it, when he deprecated the popular belief in the painfulness and incurability of cancer. His cheering message—"If you don't allow cases to drift they are not incurable"—should do more to bring funds to his hospital than any of the now rather threadbare "palaces of pain" rhetoric, at least if the giving public are intelligent enough to support scientific common sense rather than sentimental appeals to their lower nature. Mr. Howell, the secretary, has probably experience of all the old roads to the public's heart. What are his views on the cheerful one?

### THE CHEESE-PARING IN MIDDLESEX.

It is really difficult to say whether the cheese-paring policy by which the Insurance Commissioners have curtailed the scheme of the Middlesex County Council for dealing with tuberculosis in the county is matter for regret or not. On the one hand it has come to be the fashion to assume that money is all that is required for "stamping out" the disease. Recent articles in our columns, showing the paramount need for officers with special experience of the disease and of consumptive patients from an administrative point of view, have shown the folly of such credulity. On the other hand, we can but note with fresh misgiving the growingly frequent excuse of the Insurance Commissioners, here again alleged as a motive for "modifying" the County Council's scheme, that the Exchequer grant "will not be so large" as was anticipated. We fear that the Exchequer grant for the treatment of tuberculosis is not the only grant in aid, or service in kind, which is proving "not so large as was anticipated" by insured persons and others who have expected promised benefits to mature into facts. With the most solemn promise of free choice of doctor filched from them at the eleventh hour, with the callous delays which undoubtedly have been due to scamped organisation, the patient with an income of under £160 per annum, particularly if he happens to suffer from pulmonary tuberculosis, is in a piteous plight indeed just now. What are the proposals on which the Middlesex County Council has been able to decide? Briefly: The revised scheme provides for five main dispensaries at a cost of £350 each, five tuberculosis officers at £500 a year each, and two assistants at £300 each. For hospital and observation beds the capital cost to the county for 150 beds is estimated at £9,000. For sanatorium treatment the capital cost to the county of 200 beds is estimated at £12,000, and the annual charge for maintenance is £16,159, of which sum a part only will fall upon the county. While the grass is growing the steed continues to starve, and if the new appointments thus created are given to any but men with special experience, the wrong food will be given when the grass does grow at last. No provision is made for domiciliary treatment.

### THE KING'S FUND AS THE TRUSTEE OF EFFICIENCY.

A melancholy interest, as a nineteenth-century writer would say, attached to last week's meeting of the governors' court of King's College Hospital, for it was the last that will ever be held at the old hospital site in Portugal Street, Lincoln's Inn Fields. Of course, this merely means that the new buildings at Denmark Hill are nearly finished, and indeed the committee hopes to move into them towards the end of September, and proposes to close the present hospital in July. Another fact deserves to be placed on record, that, namely, King Edward's Hospital Fund for London has contributed £48,000 towards the erection of the new buildings. Its grant to the Removal Fund last year was £5,000; a fact which we emphasise because its solid support of deserving institutions is not so prominently quoted as any rulings which may happen to be inconvenient to any of the institutions which is ready to benefit from its purse. It is worth reminding all the institutions within the purview of the Fund, that in the first place, and beyond everything else, it is the trustee of an efficient and, if necessary, exacting standard for the voluntary hospitals of London, and that it is a cash-producing agency only in the secondary, if inevitable, sense of gaining by its resources the right to maintain this standard amid the fashions or panics of the hour.

### THE NOMENCLATURE OF CONDEMNATION.

New abuses require new names to brand them. The crop of the former, under the Insurance Act, has already produced a nomenclature. "Panel servitude" is the classic example which The Hospital, if not the first to christen, was the first to put into current every-day use. We have now to thank Dr. Tanner, medical officer of health for Farnham Rural District Council, for adding to the above vocabulary, the term "clerical officers of health," which aptly defines the new "status" which is being conferred on medical officers of health by the amount of time now demanded of them for clerical work in connection, for example, with keeping the records of each case of phthisis notified. For this purpose, new and elaborate books, Dr. Tanner reminds us, have been issued, entailing "entries under between sixty and seventy headings for each case." Now, a medical officer of health is the official of a public body, a body, that is, with staff, offices and resources at its command. For clerical work the supervision of the medical officer is needed; for keeping the books he should have adequate assistance. Every medical officer of health is a person who has been forced to grasp the need for the intelligent filing and interpretation of statistics. They are one of his chief weapons. Dr. Tanner's criticism, then, is not one of the system which has exalted statistics into a fine science; it is directed merely against the instruments at present at his command for the adequate diagnosis and treatment of them. His addition to the nomenclature of condemnation will, we hope, be as widely appreciated as was Pope's "tenth transmitter of a foolish face" in another kind.

### THE NEW MATRON AT NEWCASTLE.

Miss Esther Florence Corser Brown has been appointed matron and superintendent of nurses at the Royal Victoria Infirmary, Newcastle-on-Tyne, on the retirement of Miss Lucy Wilson Wamsley, who has accepted an appointment as lady inspector under the Local Government Board. Miss Brown has had a very varied and extensive experience of nursing work. She was trained at the Warneford, Leamington, and South Warwickshire Hospital during the first four years of her career. She next went as assistant matron to the Royal Portsmouth Hospital for two years, and after that she spent five years on the private staff of the Royal Sussex County Hospital, Brighton. Then Miss Brown became assistant matron to the Cancer Hospital, London, for two years; next holding the office of Secretary to the Army and Navy Male Nurses Co-operation for six months, at the end of which time she went to Newcastle as housekeeper-sister. She held the latter office for one year, and was then appointed assistant matron to the Royal Victoria Infirmary, where she has been for five years. Miss Brown has a pleasant personality, is active, strong, highly intelligent, vigorous, and deeply interested in her work. We congratulate her upon her selection to this most important office, and wish her complete success in the discharge of the arduous duties which will now devolve upon her.

### THE RED CROSS SOCIETY'S PASSING OPPORTUNITY.

There is, of course, nothing like the end of a war for making people temporarily alert to the necessity of preparing for its outbreak, and thus the Red Cross Society has been able, if not forced, to develop more than one important side of its work. Take, for instance, the voluntary aid detachments, and the need for instructing would-be officers, in times of peace, in those duties that will mainly, though by a wise latitude not solely perhaps, be expected of them in time of war. The need for giving such instruction with proper seriousness and sufficient *éclat* has led the Society to open a school of instruction which has now actually started its courses at the Polytechnic in Regent Street. The lively energy with which the Balkan War has infected such studies has found expression here in an attempt to give equal prominence to both theoretical and practical sides of the training. The course is to be under the direction of Colonel James Cantlie, who should be quite equal to ensuring permanent life to a school which necessarily it will require constant enthusiasm to maintain when the reason for its creation has been forgotten—that is to say, almost at once; by so much has daily journalism shortened the memories of the public all over the world. The storm of indignation which would probably greet us were we to name, as we could so readily, three very prominent events of the last five years which have been quite forgotten, would only be the stirrings of an uneasy conscience forced to face what many would find an unpalatable truth. So we refrain from examples.

### AN INFIRMARY SUPERINTENDENT RESIGNS.

Dr. J. Breward Neal, M.R.C.P. (Edin.), medical superintendent of St. John's Hill Infirmary, Wandsworth, has resigned his position. Dr. Neal has been for forty-two years an officer in the public service, and for thirty-four years has occupied his present post. Previous to the erection of the new infirmary, Dr. Neal had to deal with an institution which was constantly overcrowded, at times over 800 patients being under his charge. In addition, he had to contend with the disadvantages attending an old and out-of-date infirmary, where, during these trying days, Dr. Neal's enthusiasm for his work and his wide sympathy for the poor helped him to surmount the difficulties. The Guardians have placed on record their high appreciation of his skill, patience, and arduous work.

### RAISING THE SALARY OF MEDICAL OFFICERS.

The Bermondsey Borough Council has decided to increase the salary of Dr. R. K. Brown, B.A., M.D., D.P.H., medical officer, from £600 to £700 by two annual increments of £50. It is somewhat remarkable that in the riverside borough, through which passes the bulk of the food supply of the Metropolis, the salary of the medical officer should be considerably lower than that of the great majority of the metropolitan boroughs. Dr. Brown, in addition to the responsibilities attending the inspection of food imported at the wharves, has a difficult borough to deal with. Its population is poor, and there is an abundance of slum property, but he has endeavoured with remarkable success to induce not only the Borough Council but the London County Council to clear away much dilapidated property. The increase in the salary, which was fixed twelve years ago, is a well-merited tribute to his energies.

### GRATUITIES FROM PATIENTS.

We referred recently to the allegations of an Acton councillor that the staff of the Acton Urban District Council Isolation Hospital had received gratuities from patients. Although requested to attend a meeting of the appropriate committee to substantiate his assertions he did not do so, whereupon the committee very properly again demanded proof of his allegations. The councillor in question, who some time ago came into conflict with the hospital staff, has now informed the isolation hospital committee, by letter, that he does not consider any good purpose would be served by divulging the names of the persons involved in the allegations, and that the recipients of the gratuities have now left the Council's employment. He mentioned the matter to emphasise the fact that certain people were willing to give something for looking after their children, and not as a reflection on the hospital staff. The committee gave instructions for the letter to be printed *in extenso* in their minutes. When will people realise that to make allegations without adding names and details is like compounding a felony (which may be done from the humanest motives, as when a man who has evidence of a theft informs the thief that he will not prosecute if the stolen goods are returned before a given date), and is a piece of folly almost criminal in itself. For a man so destitute of responsibility as to act in this way is a danger to society—and what worse can be said of the blackmailer, the murderer, or the thief?

### JUDICIAL HANGING A RELIC OF BARBARISM.

A well-informed letter on the above subject, from the pen of Lieutenant-Colonel Marshall, L.R.C.S., appears in the current number of *The Lancet*; it confirms an impression many have had—namely, that the cause of death in executions by hanging is not always dislocation or fracture of the cervical vertebræ. Asphyxia or injury to the soft parts seems much more common than should be. The latter *contretemps*, indeed, can hardly be excluded as a possibility even by the adoption of Colonel Marshall's suggestion of the sub-mental knot. But if hanging is to be retained as a mode of capital punishment, there certainly seems a case for investigation to see whether better results are not obtainable. It is certainly crude practice to leave important details to be settled by hangmen, and we remember once hearing a rather blood-curdling story which bears out the evidence Colonel Marshall adduces as to the hit-or-miss methods of these sinister officials. This particular one stated that a man by holding his breath became heavier, and would therefore fall with more momentum. Accordingly, before he pulled the bolt he would whisper to his subject, "Hold your breath"; and one unfortunate replied forcibly that he only wished he could. Truth to tell, hanging is a barbarous method of exacting the extreme penalty long overdue for supersession. A much more humane one in every way would be the lethal chamber, because by fitting the condemned cell itself with the necessary apparatus, death could be inflicted, after a reasonable period indeed, but not at any fixed time known to the culprit (perhaps even during sleep), and thus without exciting acutely that *timor mortis* which man alone of the animal order has to bear. Those who have seen the untroubled resignation of hopeless invalids who know they have to die soon but not at what exact instant—even invalids who owe their malady to their own misdeeds—can understand how the mental agony of a Fagin in Newgate, or that better described in "The Ballad of Reading Gaol," might be mercifully spared. Nor would this method entail skilled or semi-skilled assistance, or processions, pinioning, struggles and revolting scenes and so forth. Prison medical officers and chaplains come under the wide denomination of institutional workers, and there is no need for even their steady nerves to be tried unnecessarily.

### A VETERAN MENTAL HOSPITAL WORKER.

After this week the London County Council will lose some of its most enthusiastic workers on behalf of the London mental hospitals. The announcement that Sir John McDougall is not seeking re-election for Poplar means a particularly severe loss to the Mental Hospital Committee, of which he was one of the energetic members. Sir John was one of the few members of the Council who had sat continuously since that body was first created in 1889, and he has always taken a deep interest in the mental hospitals, as is instanced by the fact that he was the only member of the Committee to serve on every one of its fourteen sub-committees. Among the other members of the Committee who did not seek re-election to the Council were Mr. T. C. E. Goff, Mr. J. W. Lorden, Mr. J. May, and Mr. W. Whitaker Thompson, the vice-chairman of the Committee and a former chairman of the Council.

### SANATORIUM BENEFIT AND CALLOUS CRUELTY

A fortnight ago we adverted in these columns on the way in which the London Insurance Committee administers sanatorium benefit. The case then quoted, and another concerning which Mr. Touche, M.P., has lately sent details to the press, seem to show that far too much is left to the discretion of clerks whose idea of dealing with serious sickness is the routine despatch of stereotyped forms, usually after considerable delay. The only alternative to this supposition appears to be a certain callous and calculated cruelty to the insured, of which we should be very sorry to suppose that any members of the Committee are knowingly or purposely guilty. In this latter case, as in many others, there seems to have been a deliberate attempt to prevent insured persons from exercising a right which the Act gives them, that of applying for permission to make their own arrangements for medical benefit. Form "Med. 21" has been deliberately suppressed in the case of the unfortunate man in whose hard case Mr. Touche is interested; and his application for it, sent in two months ago, has not yet been answered. The fact would seem to be that the Insurance Committee made up its mind to prevent these applications being sent in as far as possible, as part of its campaign against the British Medical Association; and to refuse them whenever possible. This manœuvre may be good tactics from a polemical point of view; but it entails very gross injustice and hardship to at least a few of the insured, as well as lesser degrees of prejudice to a good many others. Unfortunately, the Committee's instructions to its clerks have been so explicit and rigid that episodes like that to which Mr. Touche alludes have followed. No doubt the Committee did not foresee them, and, as in this case, will take steps to set matters right, but no plea of want of foresight can be held to absolve them from the responsibility of their policy, which only shows that the composition of the Insurance Committees, as has all along been foretold, is such as to deprive the insured of all guarantee that their best interests will be properly considered.

### PUBLIC ANALYSTS AND INSURANCE BURDENS.

The additional work entailed by the National Insurance Act is shown by the experience of Mr. Cyril Dickinson, B.Sc., F.I.C., F.C.S., the public analyst for the borough of Southwark. Owing to the large increase in the number of bacteriological specimens to be examined, especially since the Act came into force, the number of ordinary samples analysed has had to be reduced, and in order to cope with the difficulty the Borough Council has empowered the chairman of the Public Health Committee to send the bacteriological specimens, which apparently cannot be properly dealt with in this department, to the Clinical Research Association. It is computed that there has been an increase of over 100 per cent. in the number of bacteriological specimens sent in for examination since the advent of the Insurance Act. Not only has the Act in this way interfered with the analysis of food samples, but has entailed a great expenditure on the Borough Councils, for which they get no relief under the Act. We let the Act's abuses speak for themselves; they cry aloud, and comment is as needless as stringent amendment of the Act is inevitable.

### THREE RADICAL DOCTORS ADVISE THE REST.

The defeat of the British Medical Association, as everyone knows, over the panel system was primarily the result of the activities of a small set of doctors who perceived that the cause of the Liberal Party was in some danger through the revolt of the profession against the Chancellor's schemes. Not content with this successful piece of party tactics, it would seem that the same set apprehend that the revolt is only scotched, not killed —a fear which has certainly a good deal of foundation. It might be thought that the profession is little likely to accept the advice or the optimistic assurances of these particular counsellors; but the fine phrases and confident assertions in which the advice is conveyed may possibly mislead. By all means let us have an amending Act as soon as possible, for it is badly wanted. But it must be an Act passed this time only after proper discussion, and not as merely a move in the game of party politics. When the sections of it which affect medical benefit are taken in hand, the opinions of secessionists are hardly likely to prove satisfactory to the bulk of the profession.

### PRESENTATION TO A PUBLIC DISPENSER.

There was an excellent attendance of institution dispensers at the annual meeting of the Association of Certified Dispensers, which was held lately in the famous and ancient Court Room of the Apothecaries Hall. The Clerk of the Society of Apothecaries was present, and addressed the meeting. Mr. Montagu Smith, Lewisham Infirmary, was re-elected Chairman, and Mr. A. L. Anderson, Whitechapel Infirmary, who had the pleasure of declaring a balance in hand of over £93, was asked to continue as Treasurer. Mr. Albert Howell, M.P.S., to whose recent success we referred in an earlier issue, resigned the post of Hon. Secretary, and was presented with a gold watch as a memento of his efforts on behalf of the Association. He was also elected an honorary member for life. Mr. F. E. Trayner, Hackney Infirmary, was created Hon. Secretary in his stead. The Association, in its annual report, regretted that the Pharmaceutical Society had not yet acted upon the clause in the last Poisons and Pharmacy Bill, which empowers them to accept the Hall-certificated dispensers as pharmacists without further examination, and foreshadowed the presentation of a Bill before Parliament dealing with dispensers and their difficulties. What have the dispensers to say to this?

### THE HOSPITAL SUNDAY FUND MEETING.

In our correspondence column will be found a further letter from the Rev. Lionel S. Lewis. We printed a letter from this gentleman in our issue of the 22nd ult., which occupied more than a column, omitting only, as we stated in a note, a repetition of points in Mr. Lewis's speech which we had reported fully in The Hospital of February 15. But Mr. Lewis sent us another long letter full of repetitions for which we had not space, so we offered to print a shorter letter from him which appears in the present issue. We mention these circumstances to enable our readers to judge what the state of this correspondent's mind must be when, in a letter dated 27th ult., he characterises our actions just indicated as follows: "No words of mine will describe what I feel about your own action of commission and omission, but discourteous, mistaken, and unfair as I think it, I must endure it. It is, I think, unthinkable that you should allow Mr. Holland's attack on me, and give me no, or an inadequate, reply."

### THIS WEEK'S DRUG MARKET.

The article which is attracting chief attention at the moment is cod-liver oil, the price of which is steadily advancing owing to the comparatively poor results of the Norwegian fishing; the yield of cod-liver oil, up to the present, is less than half the yield at the same time last season, and, unless a considerable improvement sets in, prices are likely to continue to advance. The value of opium has receded and very little business has been done; the price of morphine also tends lower, but there has been no alteration in quotations for codeine since the reduction made a fortnight or so ago. There is no alteration in the position of quinine, and up to the present there has been no official confirmation of the statement that the agreement between the cinchona planters and the manufacturers of quinine has been signed. Ipecacuanha tends to advance in price. Jamaica sarsaparilla is dearer, as also are cardamoms. Sugar of milk is cheaper. A reduction in the price of cocaine would not come as a great surprise.

